# Screening Foster Children for Mental Disorders: Properties of the Strengths and Difficulties Questionnaire

**DOI:** 10.1371/journal.pone.0102134

**Published:** 2014-07-09

**Authors:** Stine Lehmann, Einar R. Heiervang, Toril Havik, Odd E. Havik

**Affiliations:** 1 Department of Clinical Psychology, Faculty of Psychology, University of Bergen, Bergen, Norway; 2 Regional Office for Children and Family Affairs, Region South, Tønsberg, Norway; 3 Institute of Clinical Medicine, University of Oslo, Oslo, Norway; 4 Division of Mental Health and Addiction, Oslo University Hospital, Oslo, Norway; 5 Uni Research, Uni Health, Regional Centre for Child and Youth Mental Health and Child Welfare, Bergen, Norway; McGill University, Canada

## Abstract

**Background:**

High prevalence of mental disorders among foster children highlight the need to examine the mental health of children placed out of home. We examined the properties of the Strengths and Difficulties Questionnaire (SDQ) in screening school-aged foster children for mental disorders.

**Methods:**

Foster parents and teachers of 279 foster children completed the SDQ and the diagnostic interview Developmental and Well-Being Assessment (DAWBA). Using the diagnoses derived from the DAWBA as the standard, we examined the performance of the SDQ scales as dimensional measures of mental health problems using receiver operating characteristic (ROC) analyses. Recommended cut-off scores were derived from ROC coordinates. The SDQ predictive algorithms were also examined.

**Results:**

ROC analyses supported the screening properties of the SDQ Total difficulties and Impact scores (AUC = 0.80–0.83). Logistic regression analyses showed that the prevalence of mental disorders increased linearly with higher SDQ Total difficulties scores (X^2^ = 121.47, *df* = 13, *p*<.001) and Impact scores (X^2^ = 69.93, *df* = 6, *p*<.001). Our results indicated that there is an additive value of combining the scores from the Total difficulties and Impact scales, where scores above cut-off on any of the two scales predicted disorders with high sensitivity (89.1%), but moderate specificity (62.1%). Scores above cut-off on both scales yielded somewhat lower sensitivity (73.4%), but higher specificity (81.1%). The SDQ multi-informant algorithm showed low discriminative ability for the main diagnostic categories, with an exception being the SDQ Conduct subscale, which accurately predicted the absence of behavioural disorders (LHR− = 0.00).

**Conclusions:**

The results support the use of the SDQ Total difficulties and Impact scales when screening foster children for mental health problems. Cut-off values for both scales are suggested. The SDQ multi-informant algorithms are not recommended for mental health screening of foster children in Norway.

## Introduction

The high prevalence and comorbidity of mental disorders in foster children [1]–[3] highlight the need to examine the mental health of children entering foster homes. However, child welfare services often have limited competence and resources for conducting in-depth assessments of mental health. Therefore, shorter screening tools may be useful as a first step in identifying children in need of further specialised assessments. We examined the screening properties of the Strengths and Difficulties Questionnaire (SDQ) [4] with a sample of school-aged foster children in Norway.

The SDQ is a brief mental health questionnaire measuring symptoms and impairments in the child’s daily life. Both a Total difficulties scale and an Impact scale may be considered dimensional measures of mental health [5]. Used this way, the SDQ Total difficulties score has shown good predictive ability in community samples in Britain (n = 18,415, of whom 983 had a mental disorder) [5], Sweden (n = 478, of whom 221 were clinical cases) [6], and the US (n = 1.0,367, where 9% were high scorers) [7], and in British looked-after children (n = 1391, of whom 38.6% had a mental disorder) [8]. The Impact score has also been found to be a strong predictor of mental disorders in community samples (n = 4,479, where 7% had a mental disorder) [9], service use in child welfare samples (n = 292, where 29% of these had contact with mental health care) [10], and to discriminate well between a community (n = 467) and clinical sample (n = 232) [11].

By combining the SDQ Symptom scores and the Impact score from different informants, multi-informant algorithms have been developed to estimate the probability that a child has a mental disorder [12]. In Britain, these algorithms have demonstrated acceptable levels of accuracy when predicting the type of disorder in a clinical sample (n = 101, of whom 74% had a mental disorder) [12], and in a sample of looked-after children with mental disorders (n = 539) [13]. In a community sample, these algorithms adequately discriminated between children with (n = 698) and without (n = 2.286) mental disorders, but were not suitable to discriminate between specific types of disorders [14]. In Norway, the algorithms have shown high sensitivity and specificity when screening children with chronic physical illness (n = 559, 11% high scorers) for Any mental disorder and disorder subtype [15]. However, this finding has not been confirmed in youth who have been referred to community mental health services (n = 286, of whom 66% had a mental disorder) in Norway [16].

R. Goodman, Renfrew, et al. [12] state that (the SDQ) “algorithms are… likely to work best in the sample on which they are developed” (p. 130); therefore, it is important to study the SDQ predictive algorithms in the settings in which they are to be used [17]. According to Goodman and Scott [18], the rather narrow range of problems measured by the SDQ limits its suitability in samples with broad psychopathology and high comorbidity. However, the SDQ is currently implemented as part of the annual follow-up of looked-after children in Britain [8]. Given that populations and child welfare systems differ substantially across societies [19], there is a need to examine the screening properties of the SDQ with foster children outside of Britain.

The present study examined the screening properties of the SDQ for categories of mental disorders in school-aged foster children in Norway. The following research questions were addressed: How well do the Total difficulties scale and the Impact scale discriminate between foster children with and without mental disorders? Can optimal cut-off values for use of the SDQ with foster children be recommended? Do the SDQ scales have equal validity across the full continuum of severity? Previous studies have demonstrated good predictive values for both the Total difficulties scale and the Impact scale, yet these scales have always been analysed separately. Will a combination of scores from the Total difficulties scale and the Impact scale yield additional predictive value? How accurate are the UK-based multi-informant algorithms for predicting mental disorders in foster children in Norway?

## Methods

### Measures

The SDQ is a 25-item mental health questionnaire for 3- to 16-year-olds that may be completed by parents and teachers, and as a self-report beginning at the age of 11 years [20]. The SDQ, originally developed in English, is currently available for downloading in 75 authorized translations from its official website run by Youthinmind (http://www.sdqinfo.org/). The SDQ consists of a prosocial subscale, a peer problems subscale and three symptom subscales, measuring Emotional symptoms, Conduct problems and Hyperactivity-Inattention symptoms. Each subscale consists of five items that are rated on a scale (0–1–2), providing a total score range of 0–10. A Total difficulties score is computed by summing the three symptom and the peer problem subscales, giving a total score ranging from 0–40. The two-page version of the SDQ also includes an Impact scale, measuring distress to the child and the interference of symptoms and problems in the child’s daily life [11]. The parent version of the Impact scale consists of 5 items, providing a total score range of 0–10, whereas the teacher version consists of 3 items, providing a total score range of 0–6. In a recent review of 18 studies concerning the psychometric properties of the SDQ [21], the SDQ was found to have a satisfactory internal consistency, test-retest reliability and inter rater agreement. The current five factor structure was supported by 15 of the 18 reviewed studies, two of these 15 studies presenting data from Norwegian community samples.

The multi-informant algorithms combine scores from the three SDQ symptom subscales and the Impact scale when these scales have been completed by at least two types of informants [12]. The algorithms estimate the following probabilities for the presence of a disorder: Unlikely, Possible and Probable. Independent estimates are provided for Emotional, Behavioural and Hyperactivity-Inattention disorders, and an overall estimate is provided for Any mental disorder.

The DAWBA [22] is a structured interview for the diagnostic assessment of mental disorders that may be rated according to the Diagnostic and Statistical Manual of Mental Disorders (DSM-IV) [23], or the International Classification of Diseases (ICD-10) [24]. The DAWBA may be completed by parents or caregivers, and children can complete it themselves beginning at the age of 11. There is also a shorter teacher version. Trained clinicians rate the interviews after reviewing all of the information from the informants, which is presented through a separate scoring program. The DAWBA adequately discriminates between children from community and clinical settings [22] and generates realistic prevalence estimates for mental disorders when used in public health services [25], [26]. The SDQ has been validated against the DAWBA in a number of studies [5], [8], [9], [13]–[17].

### Procedure

The data collection started on September 1^st^ 2011, and lasted until the end of February 2012. In this prospective study, eligible participants were foster children between the age of 6 and 12 years who had lived for at least 5 months in foster homes in the 63 municipalities encompassed by the Southern Regional Office for Children Youth and Family Affairs (BUFETAT), following legally mandated placement. According to the central register of BUFETAT, a total of 391 children were eligible in the 63 municipalities. Information letters were sent to the head of each municipal child welfare office. The office heads were asked to review the list of foster children from the central register, and add potentially eligible children, if any; to those in the register. This search process identified 28 additional eligible children. Twenty children who had been returned to biological families or who had been adopted were removed from the list. Another three children were deemed ineligible because of serious neurological disabilities. The final number of eligible children was therefore 396. The municipal child welfare offices were asked to provide contact information for schools and teachers of these children.

**Figure 1:**
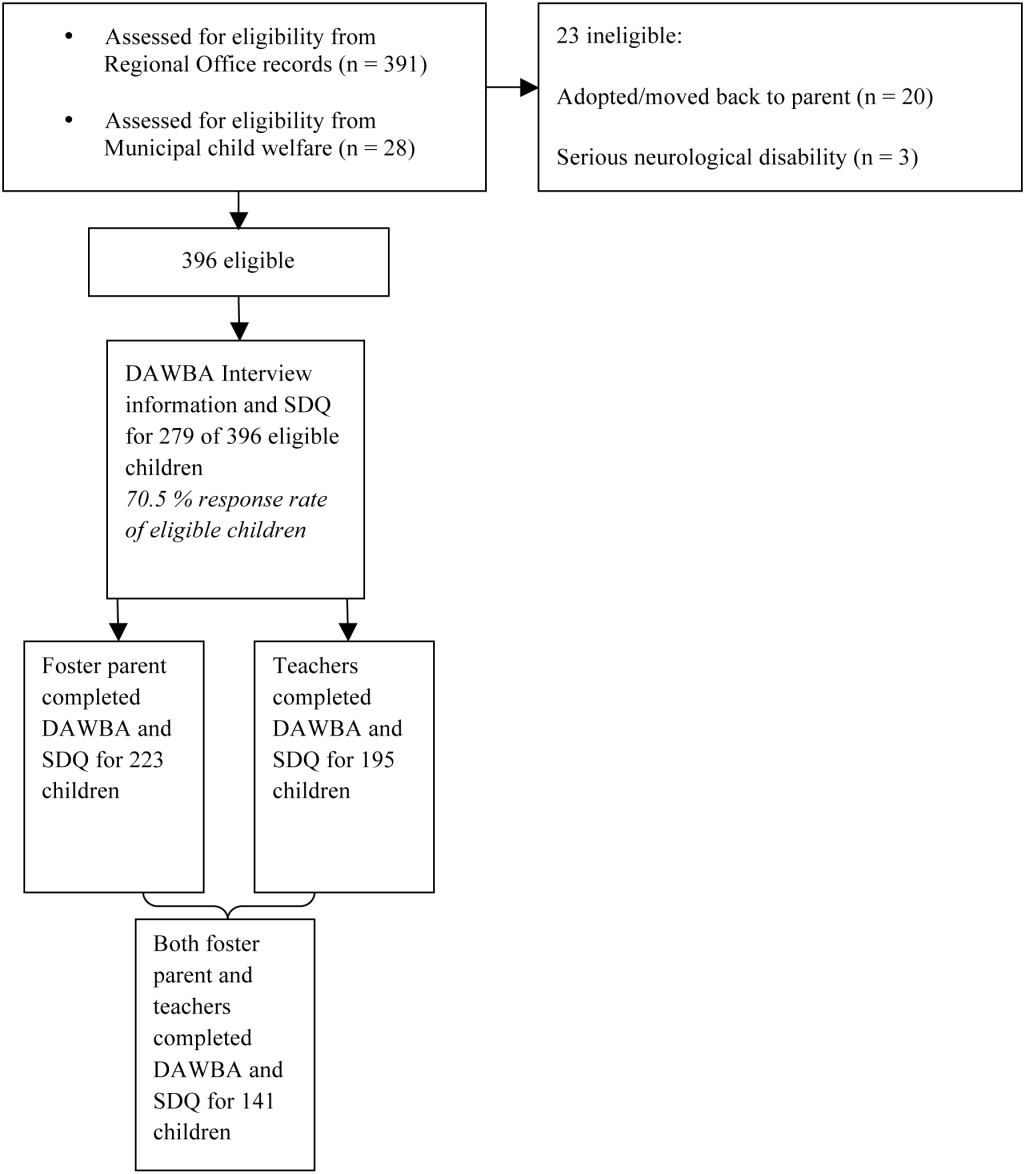
Flow chart of data collection.

Foster parents received a postal letter with detailed information about the study, and instructions on how to complete the SDQ and DAWBA interview online. They were also asked to return contact information for the children’s school and teacher. In total, contact information was obtained for 307 teachers, who were then contacted by postal mail and asked to complete SDQ and DAWBA interview online. The data collection is illustrated in figure 1.

The first and second authors, both specialists in child mental health, rated the DAWBA according to the DSM-IV criteria [23] and were blind to the SDQ scores. All available DAWBA information from both foster parents and teachers were used in the diagnostic assessment. For the present analyses, mental disorders were grouped into the following categories: Any mental disorder (includes all diagnoses), Emotional (i.e., Depression and Anxiety), Behavioural (i.e., Conduct and Oppositional Defiant disorders) and Attention Deficit/Hyperactive disorders (ADHD). Further details regarding diagnostic ratings are reported in Lehmann et al. [3].

### Ethics

The Regional Committee for Medical and Health Research Ethics for West Norway approved this study. In accordance with Norwegian ethics requirements, assent was obtained from children who were at least 12 years old. According to Norwegian legislation, foster parents do not have the mandate to consent on behalf of their foster children. The study were therefore reviewed by the Ministry of Children, Equality and Integration, who provided caseworkers, foster parents and teachers with exemption from confidentiality for the current study. The study is reported in compliance with the STARD guidelines [27].

### Study Sample

The study sample, hereafter referred to as the “All data” sample comprised 279 of 396 eligible children (70.5%), such that at least one informant, i.e. a foster parent or teacher, had completed the SDQ and DAWBA.

Analyses of the SDQ Total difficulties scale showed similar predictive values for foster fathers (n = 103: AUROC = .86, *p*<0.001, 95% CI .79–.93) and foster mothers (n = 201: AUROC = .84, *p*<0.001, 95% CI .78–.89). Therefore, we combined foster fathers and foster mothers into one group of informants, hereafter referred to as the “caregivers” (n = 223), prioritizing information from the foster mothers when available.

For the multi-informant algorithms, we used data from a subset of children who had their SDQs completed by caregivers and teachers (n = 141), hereafter referred to as the “Two informants” sample.

### Statistical Analysis

We used SPSS version 19 for Windows for data analyses, with the exception of the receiver operating characteristic (ROC) analyses, which were conducted using STATA 12.

#### The Total Difficulties and Impact scales

We conducted ROC analyses on the Total difficulties scale, the three symptom subscales and the Impact scale. Area under the receiver operating characteristics (AUROC) values were estimated for the scores reported by caregivers (n = 223) and by teachers (n = 195) separately.

The association between the SDQ scale scores and Any mental disorder were analysed by two separate logistic regression analyses using different definitions of the scales. In the first analysis, we estimated the relative increase in the prevalence of Any mental disorder with increasing scores on the Total difficulties and Impact scales. As in a previous study of SDQ as a dimensional measure [5], the scores from both SDQ scales were recoded into broader score categories in order to prevent unstable estimates due to the small number of children, i.e., n<10; at some scale scores. For the Total difficulties scale, scores 0 to 3 were collapsed into one single category “0–3”. For the SDQ score from 4–25, two and two SDQ scores were combined – e.g., scores 4 and 5 into “4–5”, 6 and 7 into “6–7” and so on. Scores from 26 and higher were recoded into “26+”. The original 40 steps in the scale were thus reduced to 13 categories. The same procedure was used for the Impact scale: Scores 0–10 were recoded into 6 categories, starting with 0, and then values 1 and 2 were collapsed into one category “1–2” and so on. In a second logistic regression analysis, the Total difficulties and Impact scales were treated as continuous variables in order to obtain Odds Ratios (*OR*) for mental disorders, as a consequence of a single step increase in the scales. We did run logistic regression analyses both for the recoded version and the original version of the scales.

Coordinates of the ROC curves were used to select optimal cut-off values for the Total difficulties and Impact scales. We calculated Sensitivity and Specificity, together with Positive and Negative predictive values. As these measures are dependent on the prevalence of disorder in the sample [28], we also calculated likelihood ratios (LHR), to express the probability that more children with a disorder would test positive relative to those without a disorder [29]. For more details regarding the use of LHR estimates, see Fisher et al [30], McGee [31], and Marasco, Doerfler and Roschier [32]. Predictive values were interpreted with use of Bayes theorem nomogram [33]. The added value of combining the Total difficulties and Impact scales was examined using logistic regression analyses.

#### Probabilities based on the multi-informant algorithms

Chi-square analyses were used to estimate the goodness of fit between the three probability levels derived from the multi-informant algorithms, and the prevalence of mental disorders. The three probability levels were then dichotomised into a conservative “Probable” cut-off level and a more liberal “Possible” cut-off level for receiving a positive test result. As for the Total difficulties and Impact scales, predictive values for the algorithms were estimated for the two cut-off levels separately.

## Results

For the “All data” sample (N = 279), the mean age of children was 9.0 years (*SD* 2.0), with 47.0% being female. As described in a previous report [3], 50.9% (n = 142) of the sample had one or more DSM-IV disorders, in the following categories: Emotional (24.0%), Behavioural (21.5%), ADHD (19.0%) and Reactive attachment disorders (RAD) (19.4%). The comorbidity rate was high with 63.4% of children with disorders having more than one mental disorder.

In the sub sample used to calculate accuracy for carer completed SDQs (n = 223), the prevalence of any disorder was 57.4%. In the subsample used to calculate accuracy for teacher completed SDQs, the prevalence of any disorder was 48.7%.

In the “Two informants” sample (n = 141), the prevalence of any disorder was 47.5%. The caregivers reported a mean SDQ Total difficulties score of 14.7 (*SD* 7.8), whereas the teachers reported a mean of 11.9 (*SD* 7.2, *t* = 4.8, *df* = 140, *p*<.001). The mean SDQ Impact score was 2.8 (*SD* 2.8) for the caregiver reports, and 1.8 (*SD* 1.9) for the teacher reports. As the Impact scale for foster parents comprised more items (5 vs 3 items) than the Impact scale for teachers, statistical analysis of the difference in mean score for the two samples could not be performed. No significant differences were evident between the “All data” and “Two informant” samples regarding age, gender, SDQ Total difficulties score or DAWBA disorder prevalence (results not shown).

### AUROC and Dimensional Properties of the Total Difficulties and Impact Scales

**Figure 2:**
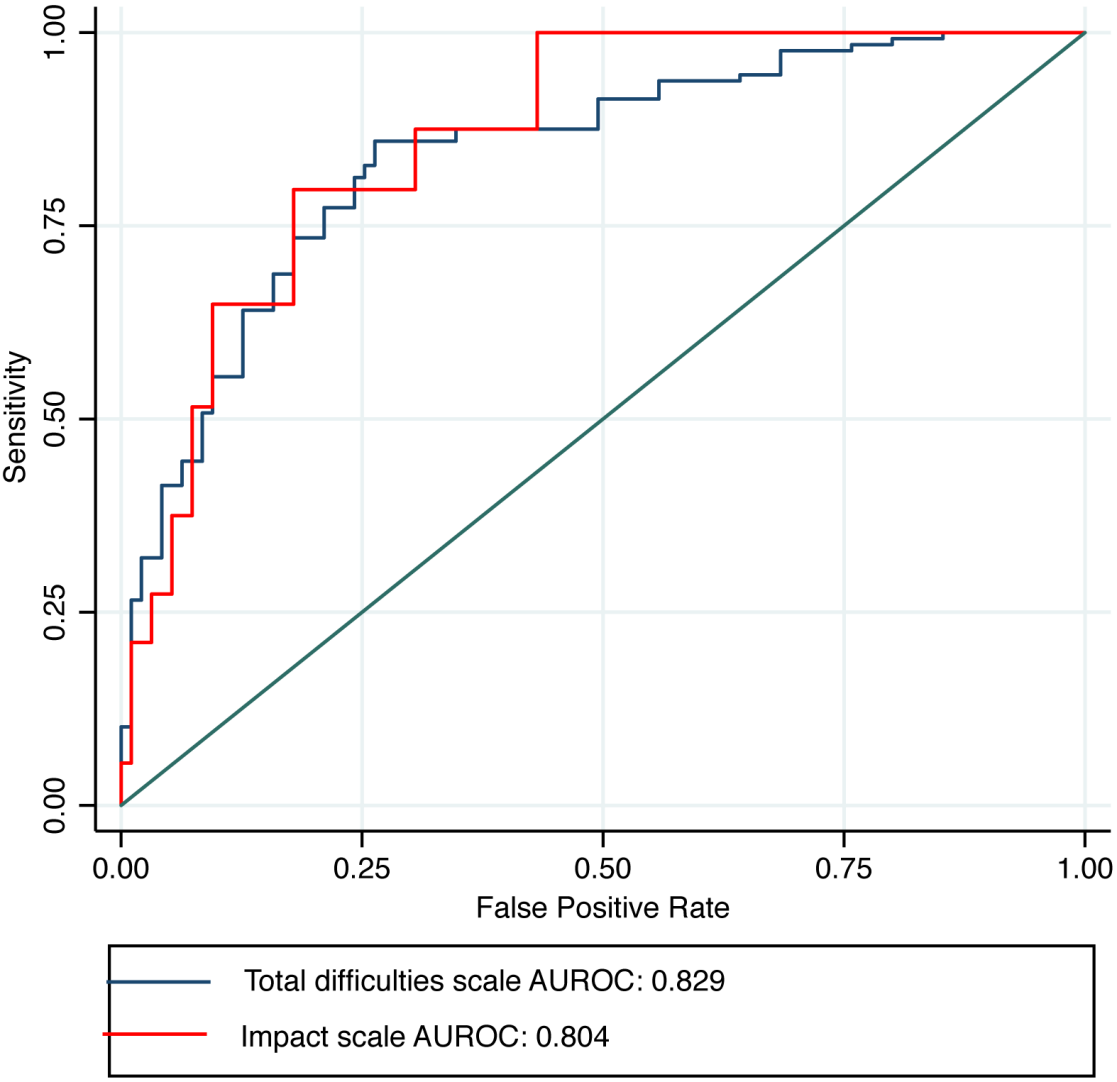
Receiver operating characteristics (ROC) curve for caregiver completed SDQ; Total difficulties scale and Impact scale (n = 223). AUROC = area under the curve.

The Total difficulties and Impact scores predicted the presence of disorders at greater than chance rates for both groups of informants (Table 1). For these scales, the results indicate excellent accuracy for caregivers and acceptable accuracy for teachers, according to criteria suggested by Hosmer Jr et al. [34]. Overall, the predictive values for the three SDQ subscale scores were comparable to those for the Total difficulties and Impact scores. Figure 2 displays the ROC curve for the Total Difficulties and Impact scales completed by caregivers (n = 223).

**Table 1:** Area Under the Receiver Operating Curve for SDQ Scales.

SDQ scales on DAWBA diagnostic groups	Caregiver SDQ (n = 223)	Teacher SDQ (n = 195)
	AUROC 95% CI	AUROC 95% CI
Total difficulties on Any disorder	.83 [.78, .88]	.77 [.71, .86]
Impact on Any disorder	.80 [.75, .86]	.75 [.68, .82]
Emotional subscale on Emotional disorder	.82 [.76, .88]	.74 [.66, .82]
Conduct subscale on Behavioral disorder	.89 [.84, .93]	.86 [.80, .93]
Hyperactive subscale on ADHD	.81 [.74, .87]	.80 [.72, 87]

**Figure 3:**
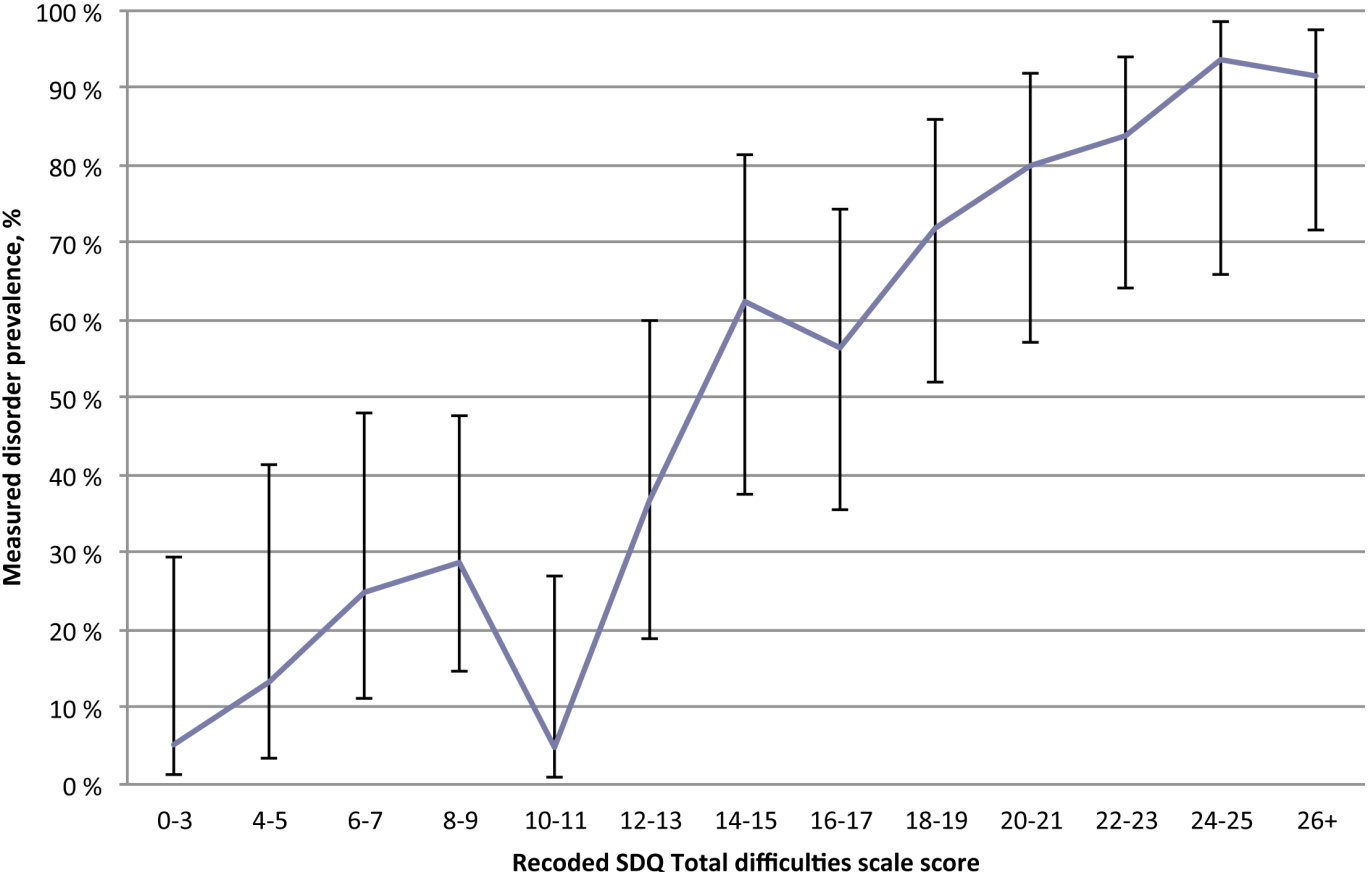
SDQ Total difficulties scale score and prevalence of mental disorders (95% CI) in foster children (N = 279).

The level of agreement between the increase in recoded Total difficulties scores and the increase in prevalence of mental disorders was strong (X^2^ = 121.47, Kendall’s tau-b.47, *df* = 13, *p*<.001) for the “All data” sample, as illustrated in Figure 3. The recoded scores of “10–11” and “16–17” represented a break in the linear trend.

**Figure 4:**
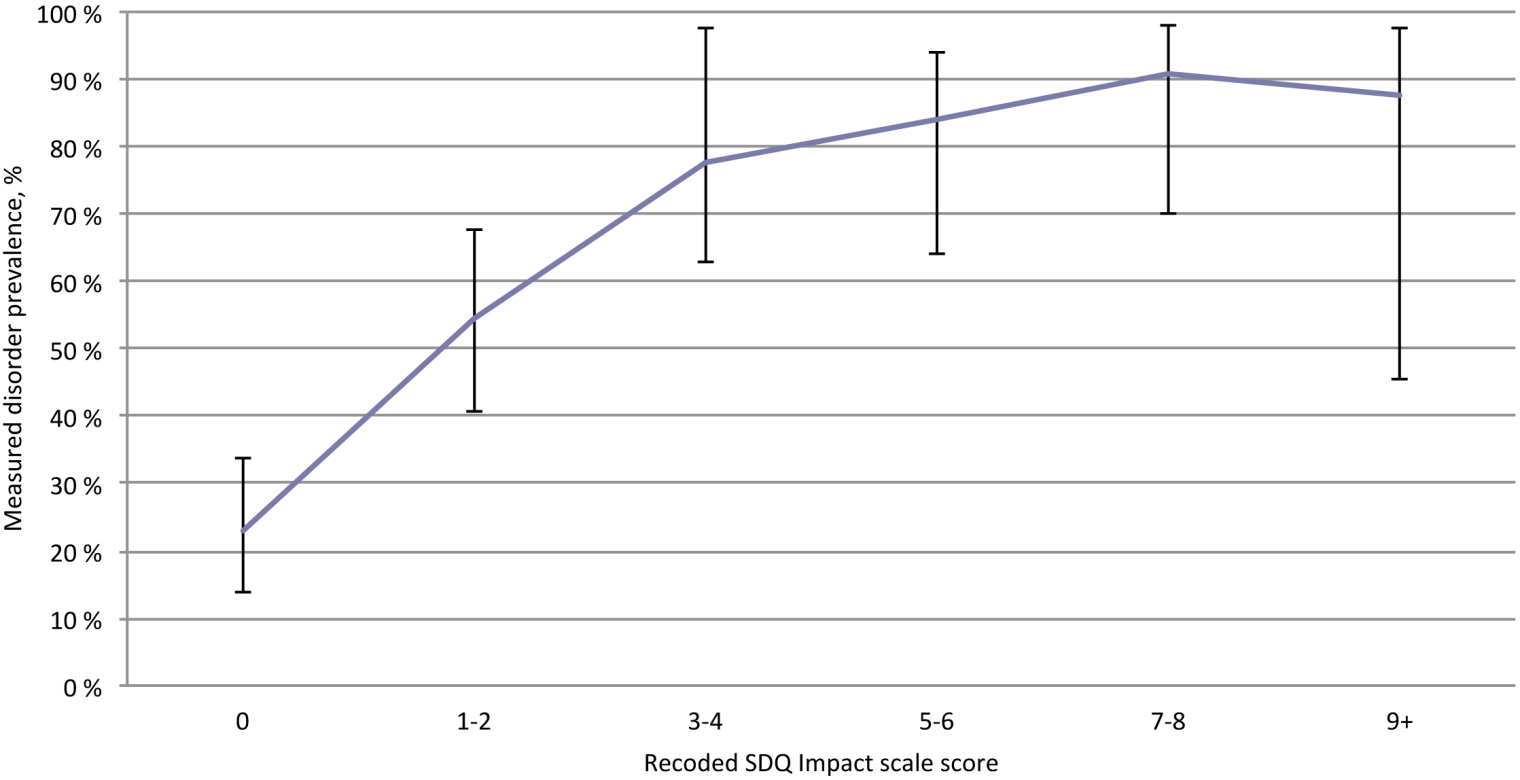
Caregivers SDQ Impact scale score and prevalence of mental disorders (95% CI) in foster children (n = 223).

An increase in the recoded carer completed SDQ Impact scores corresponded to an increased prevalence of Any mental disorder (X^2^ = 69.93, Kendall’s tau-b.46 *df* = 6, *p*<.001) (Figure 4).

In the logistic regression analyses, the Total difficulties scale and the Impact scale was entered as continuous scales to estimate the ORs for the risk for Any mental disorder related to one step increase on the relevant scale. The ORs were nearly identical for the recoded and original scale versions: Total difficulties scale: recoded: OR = 1.24 (95% CI 1.18–1.30), original: OR = 1.23 (95% CI 1.17–1.29). The Impact scale: recoded: OR = 1.68 (95% CI 1.42–1.98), original: OR = 1.69 (95% CI 1.44–1.98).

### Cut-Off Values for the Total Difficulties and Impact Scales

Table 2 presents the sensitivities and specificities of the different Total difficulties scores, which were derived from the ROC analysis. Given equal weight to specificity and sensitivity, a cut-off score of 13 is optimal for both caregivers (82.8% sensitivity, 73.7% specificity) and teachers (86.4% sensitivity, 77.3% specificity).

**Table 2:** Receiver Operating Characteristics Analyses for the SDQ Total Difficulties Scale.

Score	Any Mental disorder			
	Caregiver (n = 223)		Teacher (n = 195)	
	Sensitivity	Specificity	Sensitivity	Specificity
7	0.945	0.316	0.949	0.386
8	0.938	0.358	0.949	0.455
9	0.914	0.442	0.949	0.477
10	0.875	0.505	0.915	0.500
11	0.875	0.611	0.881	0.545
12	0.859	0.653	0.864	0.705
13	0.828	0.737	0.864	0.773
14	0.813	0.747	0.831	0.795
15	0.773	0.758	0.797	0.795

Table 3 presents the sensitivities and specificities of the different Impact scale scores, which were derived from the ROC analysis. Given equal weight to specificity and sensitivity, a cut-off score of 2 (80.0% sensitivity, 70.0% specificity) is suggested for caregiver’s SDQ, whereas a cut-off score of 1 (77.9% sensitivity, 67.0% specificity) is optimal for teacher’s SDQ.

**Table 3:** Receiver Operating Characteristics Analyses for the SDQ Impact Scale.

Score	Any Mental disorder			
	Caregiver (n = 223)		Teacher (n = 195)	
	Sensitivity	Specificity	Sensitivity	Specificity
0	1	0.000	1	0.000
1	0.875	0.568	0.779	0.670
2	0.797	0.695	0.653	0.740
3	0.648	0.821	0.537	0.850

AUROC values revealed overlapping confidence intervals for males and females, and the coordinates for the curves indicated similar cut-off points across genders.

Table 4 illustrates the distribution of cases and non-cases for test positives and test negatives according to the recommended cut-offs, for carer completed SDQ and teacher completed SDQ respectively.

**Table 4:** Children Scoring Under and Above Recommended Cut-offs, and Prevalence of Mental Disorders According to the DAWBA interview for Carer-completed SDQ (n = 223) and Teacher-completed SDQ (n = 195).

	Carer completed SDQ				Teacher completed SDQ			
	Any mental disorder				Any mental disorder			
	Yes		No		Yes		No	
	n	%	n	%	n	%	n	%
Low S	22	9.9	70	31.4	33	16.9	77	39.5
High S	106	47.5	25	11.2	62	31.8	23	11.8
Low I	26	11.7	66	29.6	21	10.8	57	29.2
High I	102	45.7	29	13.0	74	37.9	43	22.1

*Low S* = Below symptom cut-off; *High S* = Above symptom cut-off; *Low I* = Below impact cut-off; *High I* = Above impact cut-off.

As shown in table 5, we estimated the possible additive value of combining the Total difficulties and the Impact scales when interpreting the SDQ reports, using the recommended cut-off scores for both scales on SDQs completed by caregivers. With foster children scoring below the suggested cut-offs on both scales serving as a reference group, a score above the cut-off on either of the two scales increased the risk for Any mental disorder (adjusted *OR* 4.70, 95% CI 1.98–11.10, *p*<.001), predicting Any mental disorder with 89.1% sensitivity and 62.1% specificity. Scores above the cut-offs on both scales predicted Any mental disorder with 73.4% sensitivity and 81.1% specificity. Post-hoc tests revealed a significant increase in the risk for Any mental disorder for children who scored above the cut-offs on both scales compared to those who scored above the cut-off on only one of the scales.

**Table 5:** Applying recommended cut-offs for SDQ: Total Difficulties Scale and Impact Scale for Caregiver SDQs (n = 223*)*.

	Sample		Disorder			
	N	%	N	%	OR	95% CI
Low S – Low I	73	32.7	14	19.2	Reference group	
Low S – High I	19	8.5	8	42.1	3.10	[1.04, 9.04]*
High S – Low I	19	8.5	12	63.2	7.22	[2.41, 21.69]**
High S – High I	112	50.2	94	83.9	22.00	[10.18, 47.56]**

*Note: Low S* = Below symptom cut-off; *High S* = Above symptom cut-off; *Low I* = Below impact cut-off; *High I* = Above impact cut-off.

**p<.001;

*p<.05.

Table 6 shows the predictive values of recommended cut-offs for each scale of carer completed SDQs, separately and combined. The likelihood ratios indicate that a cut-off at 13 on the Total difficulties score will increase the post-test probability of any disorder to 81.0%, from the pre-test probability of 57.4%. A negative test will decrease the post-test probability to 23.0%. The predictive value of the Impact score was somewhat lower for test positive scores. Using the combination of Total difficulties and Impact score, scoring above cut-off on both scales will increase the post-test probability to 84.0%, but with a decreasing predictive value for negative tests to a post-test probability of 30.0%. By defining test positives as scoring above cut off on one of the scales, the probability of disorder will increase to only 76.0%, while test-negatives by will decrease their probability of disorder to 19.0%, from the pre-test probability of 57.4%.

**Table 6:** Properties of SDQ Total Difficulties and Impact Scales with Recommended Cut-offs for Any Disorder According to the DAWBA interview for Carer Completed SDQ (n = 223).

Carer completed SDQs	PPV	NPV	LHR+	LHR−
SDQ Total difficulties score 13+	0.81	0.76	3.15	0.23
SDQ Impact score 2+	0.78	0.72	2.61	0.29
Combined, above both cut-offs	0.84	0.69	3.88	0.33
Combined, above one cut-off	0.76	0.80	2.35	0.18

*Note: PPV = *Positive predictive value; *NPV = *Negative predictive value; *LHR+* = Positive likelihood ratio; *LHR− = *Negative likelihood ratio.

### The Multi-Informant Algorithms: Testing the Predictive Values of Two Different Cut-Off Scores

In the “Two informants” sample (n = 141), the multi-informant algorithm predicted that Any mental disorder was “Unlikely” for 32.3% of the children, “Possible” for 24.7% and “Probable” for 43.0%. The level of agreement between the SDQ algorithms’ results and the prevalence of Any mental disorder from DAWBA, as presented in table 7, was strong (X^2^ = 37.15, Kendall’s tau-b = .49, 95% CI = .35–.62, *p*<.001). A similar level of agreement was observed for the algorithmic predictions derived from the three SDQ symptom subscales and their corresponding diagnostic categories. The agreement was strongest for Behavioural disorders (X^2^ = 46.87, Kendall’s tau-b.55, 95% CI = .44–.65, *p*<.001) and somewhat more moderate for ADHD disorders (X^2^ = 27.68, Kendall’s tau-b = .37, 95% CI = .22–.51, *p*<.001) and Emotional disorders (X^2^ = 24.27, Kendall’s tau-b = .39, 95% CI = .23–.54, *p*<.001).

**Table 7:** Estimated Probability for Mental Disorders from the Multi-informant Algorithms, and Prevalence of Mental Disorders According to DAWBA.

SDQ Prediction	Observed Mental Disorders							
	Any		Emotional		Behavioral		ADHD	
	n	%	n	%	n	%	n	%
Disorder unlikely	13/48	27.1	11/87	12.6	0/71	0.0	5/67	7.5
Disorder possible	13/27	48.1	14/37	37.8	8/30	26.7	3/31	9.7
Disorder probable	55/66	83.3	11/17	64.7	22/40	55.0	28/43	46.5

Two Informants Sample (n = 141).

Table 8 presents the accuracy of the algorithms in predicting the corresponding DAWBA diagnostic groups based on the two cut-offs “Probable” and “Possible”. Sensitivity was highest when the “Possible” cut-off was used. However, this cut-off had relatively low specificity. Using the stricter “Probable” cut-off for positive cases, sensitivity declined and specificity increased. Although this latter cut-off demonstrated sufficient ability to include only those children with a disorder, the relatively low sensitivity renders this cut-off level unsuitable for screening purposes.

**Table 8:** Properties of SDQ Multi-informant Algorithms for SDQ Total Difficulties Scale and Subscales, for Corresponding Diagnostic Groups According to the DAWBA interview (n = 141).

SDQ prediction of corresponding DAWBA disorders	Sensitivity		Specificity		PPV		NPV		LHR+		LHR−	
	Prob	Poss	Prob	Poss	Prob	Poss	Prob	Poss	Prob	Poss	Prob	Poss
SDQ Total- DAWBA Any disorders	0.68	0.84	0.82	0.58	0.83	0.73	0.65	0.73	3.70	2.02	0.39	0.28
SDQ Emotional - DAWBA Emotional disorders	0.30	0.69	0.94	0.72	0.65	0.46	0.80	0.87	5.35	2.51	0.74	0.42
SDQ Conduct - DAWBA Behavioral disorders	0.73	1.00	0.84	0.64	0.55	0.43	0.92	1.00	4.52	2.78	0.32	0.00
SDQ Hyperactive - DAWBA ADHD disorders	0.71	0.82	0.80	0.55	0.47	0.31	0.92	0.92	3.51	1.82	0.36	0.33

*Note.* P*rob* = Dichotomized on Probable level; *Poss* = Dichotomized on Possible level; *PPV* = Positive predictive value; *NPV* = Negative predictive value; *LHR+* = Positive likelihood ratio; *LHR−* = Negative likelihood ratio.

Based on the LHR+ values, only the SDQ Emotional subscale with the “Probable” cut-off had the potential to identify emotional disorders without including too many false positives. Findings in a previous report [3] indicate that the pre-test probability of having an Emotional disorder is 24.0% for Norwegian foster children. An LHR+ value of 5.35 for the SDQ Emotional subscale signifies an increased post-test probability of disorder of 62.0% for Emotional disorders in children who scored above the cut-off. However, an LHR− value of 0.74 suggests that scoring below the cut-off decreases the probability of disorder only slightly, to a post-test probability of 19.0%.

Only the “Possible” cut-off for the Conduct subscale showed potential predictive usefulness, as no child scoring below this cut-off had Behavioural disorders, compared with a pre-test prevalence of 21.5%.

## Discussion

### The Total Difficulties and Impact Scales

The ability of the Total difficulties and Impact scales to discriminate between children with and without Any disorder, according to the ROC analyses, is in the upper range compared to results from previous studies on SDQ used with school-aged children [21]. Furthermore, the AUROC for these two scales revealed discriminative ability superior to that reported for Norwegian pre-school children [35], especially as measured by the Impact scale. Examining an older age group with a higher prevalence of disorders may have contributed to the present findings for foster children compared to the pre-school community sample.

Our findings regarding the screening properties of the SDQ as a dimensional measure are generally consistent with previous reports with community samples [5], [17], clinical samples [11] and looked-after children [8]. This suggests that the Total difficulties and Impact scales are appropriate for use across samples with different disorder prevalence rates. Our findings also suggest that SDQ used as a dimensional measure is valid across a continuum of severity and thereby suitable for screening purposes in foster children with a broad range of mental health problems.

One purpose of screening is to identify children who are in need of more in-depth mental health assessments. To aid in this decision, a cut-off value is often preferred. Here, the consequences of not detecting mental disorders must be weighed against the costs of extensive assessments of children who do not have a disorder. Although a cut-off of 13 on the carer-completed Total difficulties scale may provide the best balance between sensitivity and specificity, it is important to note that children with Total difficulties scores in the low range from 4 to 9 had a prevalence of disorders ranging between 13.0 and 29.0% (Figure 3).

In line with this finding, the high prevalence of mental disorders in foster children warrants a general alertness in child welfare settings. False positives may still have vulnerabilities that do not manifest until children are exposed to new situations, demands and expectations, e.g., starting school. Furthermore; one cannot rule out the possibility that false positives in this high risk group are children with substantial mental health problems, just below the requirements of diagnostic criteria. For example, in a newly reported study on mental health screening in a foster-care sample from New Zealand (N = 577), Tarren-Sweeny [36] found that a majority of false-positive children had at least one mental health score in clinical range as measured with Child Behaviour Checklist [37]. Post-hoc analyses of our data support this finding. Depending on the subscale, 52.0–88.0% of false positives were high-scorers (defined as one *SD* + above mean score using British norms). Therefore, cut-offs with higher sensitivity may be preferable, in spite of their lower specificity.

An optimal balance between sensitivity and specificity was obtained when the cut-offs for both scales were combined. Defining test positives as a score above the cut-off on one of the two scales identified 89.1% of the children with a disorder. Of the test positives, 37.9% did not have a mental disorder. The added predictive value when combining these two scales indicate that the Impact scale and the Total difficulties scale are not parallel; rather, they complement each other by measuring different but equally relevant aspects of child mental health. In high-risk samples, not only a high prevalence rate; but also a broad range of symptoms and high comorbidity may contribute to these results, which render the Impact scale equally important as the Total difficulties scale for screening purposes.

To sum up, if the main purpose of screening is to reduce the number of undetected (false negative) children with a need for more detailed mental health examination, then we recommend cut-offs at either 13+ on the Total difficulties scale or 2+ on the Impact scale to be defined as test positives. The low negative likelihood ratio for this combination indicates a decrease in post-test probability of having a disorder from 57.4% to 19.0% for test-negatives. If on the other hand, an equal emphasize on positive and negative predictive values is preferred, then test positives could be defined by scoring above cut-off on Total difficulties scale only, regardless of score on the Impact scale. We cannot recommend scoring above cut-off on both Total difficulties and Impact scale as a requirement to be defined as test positive, as 30.0% of test negatives here have a post-test probability of having a disorder. For teacher-completed SDQs, the threshold for the Impact scale should be lowered to 1+, while the recommended cut-off for the Total difficulties scale remains 13.

### The Multi-Informant Algorithms

Although estimates derived from the algorithms showed some discriminative ability (Table 7), the predictive values for the four diagnostic categories used in the present study were moderate to low, according to Fisher’s guidelines [30]. However, the algorithmic estimates for Behavioural disorders showed markedly more sensitivity compared to those for Emotional disorders.

Goodman et al. [13] found 85.0% sensitivity and 80.0% specificity for the “Probable” prediction of Any mental disorder in looked-after British children. Given that the overall rates of disorder in our sample were comparable to those of that sample; our lower sensitivity is somewhat surprising. However, a previous study of the predictive value of the multi-informant algorithms in a Norwegian clinical sample reported results similar to ours [16]. The algorithms are calculated using a fixed combination of scores, derived from a British normative sample [25]. Finnish norms for SDQ suggests a cut-off 2–3 points lower than that derived from the British norms [38], illustrating that the UK multi-informant algorithms are based on cut-offs that may not fit populations in other countries. Furthermore, when the algorithms were examined with a British clinical sample [12], the algorithms were modified by increasing the threshold for identifying emotional disorders. For both the clinical sample and the looked-after British children, behavioural disorders were reported almost three times as often as emotional disorders. By contrast, in our sample of Norwegian foster children, there were similar prevalence rates of these two disorders, with a lower rate of behavioural disorders and a higher rate of emotional disorders than in the British samples [3].

### Limitations

The statistical analyses presented for the Total difficulties scale, the Impact scale and the multi-informant algorithms are all based on dichotomous diagnostic outcomes. However, individuals differ not only in the presence or absence of a disorder but also in the severity and number of symptoms experienced, their duration and their impact on daily life [39]. In a high-prevalence sample, the size of this sub-threshold group would be larger than in the general population, which would decrease the predictive value of a screening instrument with a defined cut-off value.

In addition, when a sample is divided into subgroups, the sample size determines the degree of vulnerability for random errors in the values of the target variable. In our study, the relatively small sample size may have influenced the fit between the Total difficulties score and the prevalence of disorders, as illustrated in Figure 3. Here, a relatively steadily ascending curve is interrupted by sudden drops that occur at scores “10–11” and “16–17”, suggesting need for caution when interpreting our results. The relatively large confidence intervals add to this reservation. Nevertheless, Chi-square analyses with corresponding *OR*s suggest that there is a relatively good correspondence between the increase in SDQ scores and the prevalence of mental disorders. Furthermore, the nearly identical ORs for the recoded and original version of the Total difficulties and the Impact scales support the validity of SDQ used as a dimensional measure across a continuum of severity.

### Clinical Implications

The good fit between the increased SDQ scores and the prevalence of disorders suggests that the SDQ is a useful measure for guiding service plans and for comparing child welfare groups with regard to intervention needs. Furthermore, the use of brief mental health questionnaires, such as the SDQ, may both improve communication between child welfare and mental health services, and facilitate the description of children’s needs across these relevant services.

If a cut-off for further assessment is preferred, we recommend the use of an interpretation that is based on a combination of the Total difficulties score and the Impact score. Our findings suggest that either a Total difficulties score of 13+ *or* an Impact score of 2+ for the carer-completed SDQ may indicate the presence of a mental disorder and warrants a follow-up with the child. Based on our findings, we cannot recommend the use of the predictive algorithm to screen foster children in Norway for mental disorders.

## References

[1] Ford T, Vostanis P, Meltzer H, Goodman R (2007) Psychiatric disorder among British children looked after by local authorities: Comparison with children living in private households. British Journal of Psychiatry 190 (APR.): 319–325. 10.1192/bjp.bp.106.025023 17401038

[2] McMillen JC, Zima BT, Scott LD Jr, Auslander WF, Munson MR, et al (2005) Prevalence of psychiatric disorders among older youths in the foster care system. Journal of the American Academy of Child and Adolescent Psychiatry 44(1): 88–95. 15608548 10.1097/01.chi.0000145806.24274.d2

[3] Lehmann S, Havik O, Havik T, Heiervang E (2013) Mental disorders in foster children: a study of prevalence, comorbidity and risk factors. Child and Adolescent Psychiatry and Mental Health 7: 39. 24256809 10.1186/1753-2000-7-39 PMC3922948

[4] Goodman R (1997) The Strengths and Difficulties Questionnaire: A research note. Journal of Child Psychology and Psychiatry 38: 581–586. 9255702 10.1111/j.1469-7610.1997.tb01545.x

[5] Goodman A, Goodman R (2009) Strengths and Difficulties Questionnaire as a dimensional measure of child mental health. Journal of the American Academy of Child & Adolescent Psychiatry 48: 400–403. 19242383 10.1097/CHI.0b013e3181985068

[6] Malmberg M, Rydell A-m, Smedje H (2003) Validity of the Swedish version of the Strengths and Difficulties Questionnaire (SDQ-Swe). Nordic Journal of Psychiatry 57: 357–363. 14522609 10.1080/08039480310002697

[7] Bourdon KH, Goodman R, Rae DS, Simpson G, Koretz DS (2005) The Strengths and Difficulties Questionnaire: US normative data and psychometric properties. Journal of the American Academy of Child & Adolescent Psychiatry 44: 557–564. 15908838 10.1097/01.chi.0000159157.57075.c8

[8] Goodman A, Goodman R (2012) Strengths and Difficulties Questionnaire scores and mental health in looked after children. The British Journal of Psychiatry 200: 426–427. 22550331 10.1192/bjp.bp.111.104380

[9] Stringaris A, Goodman R (2013) The Value of measuring impact alongside symptoms in children and adolescents: A longitudinal assessment in a community sample. Journal of Abnormal Child Psychology: 1–12. 23677767 10.1007/s10802-013-9744-x PMC3755220

[10] Janssens A, Deboutte D (2009) Screening for psychopathology in child welfare: the Strengths and Difficulties Questionnaire (SDQ) compared with the Achenbach System of Empirically Based Assessment (ASEBA). European Child & Adolescent Psychiatry 18: 691–700. 19462154 10.1007/s00787-009-0030-y

[11] Goodman R (1999) The extended version of the Strengths and Difficulties Questionnaire as a guide to child psychiatric caseness and consequent burden. Journal of Child Psychology and Psychiatry 40: 791–799. 10433412

[12] Goodman R, Renfrew D, Mullick M (2000) Predicting type of psychiatric disorder from Strengths and Difficulties Questionnaire (SDQ) scores in child mental health clinics in London and Dhaka. European Child & Adolescent Psychiatry 9: 129–134. 10926063 10.1007/s007870050008

[13] Goodman R, Ford T, Corbin T, Meltzer H (2004) Using the Strengths and Difficulties Questionnaire (SDQ) multi-informant algorithm to screen looked-after children for psychiatric disorders. European Child & Adolescent Psychiatry 13: ii25–ii31. 15243783 10.1007/s00787-004-2005-3

[14] Goodman R, Ford T, Simmons H, Gatward R, Meltzer H (2000) Using the Strengths and Difficulties Questionnaire (SDQ) to screen for child psychiatric disorders in a community sample. The British Journal of Psychiatry 177: 534–539. 11102329 10.1192/bjp.177.6.534

[15] Hysing M, Elgen I, Gillberg C, Lie SA, Lundervold AJ (2007) Chronic physical illness and mental health in children. Results from a large-scale population study. Journal of Child Psychology and Psychiatry 48: 785–792. 17683450 10.1111/j.1469-7610.2007.01755.x

[16] Brøndbo PH, Mathiassen B, Martinussen M, Heiervang E, Eriksen M, et al (2011) The Strengths and Difficulties Questionnaire as a screening instrument for Norwegian child and adolescent mental health services, application of UK scoring algorithms. Child and Adolescent Psychiatry and Mental Health 5: 1–10. 21992589 10.1186/1753-2000-5-32 PMC3207884

[17] Goodman A, Goodman R (2011) Population mean scores predict child mental disorder rates: validating SDQ prevalence estimators in Britain. Journal of Child Psychology and Psychiatry 52: 100–108. 20722879 10.1111/j.1469-7610.2010.02278.x

[18] Goodman R, Scott S (1999) Comparing the Strengths and Difficulties Questionnaire and the Child Behavior Checklist: is small beautiful? Journal of Abnormal Child Psychology 27: 17–24. 10197403 10.1023/a:1022658222914

[19] Martin MJ, Conger RD, Schofield TJ, Dogan SJ, Widaman KF, et al (2010) Evaluation of the interactionist model of socioeconomic status and problem behavior: A developmental cascade across generations. Development and Psychopathology 22: 695–713. 20576188 10.1017/S0954579410000374 PMC2892802

[20] Goodman R, Meltzer H, Bailey V (1998) The Strengths and Difficulties Questionnaire: A pilot study on the validity of the self-report version. European Child & Adolescent Psychiatry 7: 125–130. 9826298 10.1007/s007870050057

[21] Stone LL, Otten R, Engels RC, Vermulst AA, Janssens JM (2010) Psychometric properties of the parent and teacher versions of the Strengths and Difficulties Questionnaire for 4-to 12-year-olds: a review. Clinical Child and Family Psychology Review 13: 254–274. 20589428 10.1007/s10567-010-0071-2 PMC2919684

[22] Goodman R, Ford T, Richards H, Gatward R, Meltzer H (2000) The Development and Well-Being Assessment: Description and initial validation of an integrated assessment of child and adolescent psychopathology. Journal of Child Psychology and Psychiatry 41: 645–655. 10946756

[23] American Psychiatric Association (2000) Diagnostic and statistical manual of mental disorders: DSM-IV-TR. Arlington, VA: Author.

[24] World Health Organization (1992) The ICD-10 classification of mental and behavioural disorders: clinical descriptions and diagnostic guidelines. Geneva, Switzerland: Author.

[25] Meltzer H, Gatward R, Goodman R, Ford T (2003) Mental health of children and adolescents in Great Britain. International Review of Psychiatry 15: 185–187. 12745331 10.1080/0954026021000046155

[26] Heiervang E, Stormark KM, Lundervold AJ, Heimann M, Goodman R, et al (2007) Psychiatric disorders in Norwegian 8-to 10-year-olds: An epidemiological survey of prevalence, riskfactors and service use. Journal of the American Academy of Child & Adolescent Psychiatry 46: 438–447. 17420678 10.1097/chi.0b013e31803062bf

[27] Bossuyt PM, Reitsma JB, Bruns DE, Gatsonis CA, Glasziou PP, et al (2003) Towards complete and accurate reporting of studies of diagnostic accuracy: The STARD initiative. Clinical Chemistry 49: 1–6. 12507953 10.1373/49.1.1

[28] Akobeng AK (2007) Understanding diagnostic tests 1: sensitivity, specificity and predictive values. Acta Paediatrica 96: 338–341. 17407452 10.1111/j.1651-2227.2006.00180.x

[29] Deeks JJ, Altman DG (2004) Statistics notes: diagnostic tests 4: likelihood ratios. BMJ: British Medical Journal 329: 168. 15258077 10.1136/bmj.329.7458.168 PMC478236

[30] Fischer JE, Bachmann LM, Jaeschke R (2003) A readers' guide to the interpretation of diagnostic test properties: clinical example of sepsis. Intensive care medicine 29: 1043–1051. 12734652 10.1007/s00134-003-1761-8

[31] McGee S (2002) Simplifying likelihood ratios. Journal of general internal medicine 17: 647–650. 10.1046/j.1525-1497.2002.10750.x PMC1495095 12213147

[32] Marasco J, Doerfler R, Roschier L (2011) Doc, what are my chances. UMAP Journal 32: 279–298.

[33] Fagan TJ (1975) Letter: nomogram for Bayes theorem. The New England journal of medicine 293: 257–257. 1143310 10.1056/NEJM197507312930513

[34] Hosmer Jr DW, Lemeshow S, Sturdivant RX (2013) Applied logistic regression: Wiley.com.

[35] Sveen TH, Berg-Nielsen TS, Lydersen S, Wichstrøm L (2013) Detecting psychiatric disorders in preschoolers: Screening with the Strengths and Difficulties Questionnaire. Journal of the American Academy of Child & Adolescent Psychiatry 52: 728–736. 23800486 10.1016/j.jaac.2013.04.010

[36] Tarren-Sweeney M (2013) The Brief Assessment Checklists (BAC-C, BAC-A): Mental health screening measures for school-aged children and adolescents in foster, kinship, residential and adoptive care. Children and Youth Services Review 35: 771–779.

[37] Achenbach TM, Rescorla L (2001) Manual for ASEBA school-age forms & profiles. University of Vermont, Research Center for Children, Youths and Families: Aseba Burlington.

[38] Borg A-M, Kaukonen P, Joukamaa M, Tamminen T (2013) Finnish norms for young children on the Strengths and Difficulties Questionnaire. Nordic Journal of Psychiatry 0: 1–10. 10.3109/08039488.2013.853833 24228779

[39] Rutter M, Sroufe LA (2000) Developmental psychopathology: Concepts and challenges. Development and Psychopathology 12: 265–296. 11014739 10.1017/s0954579400003023

